# Weak Epistasis Generally Stabilizes Phenotypes in a Mouse Intercross

**DOI:** 10.1371/journal.pgen.1005805

**Published:** 2016-02-01

**Authors:** Anna L. Tyler, Leah Rae Donahue, Gary A. Churchill, Gregory W. Carter

**Affiliations:** The Jackson Laboratory, Bar Harbor, Maine, United States of America; Washington University Medical School, UNITED STATES

## Abstract

The extent and strength of epistasis is commonly unresolved in genetic studies, and observed epistasis is often difficult to interpret in terms of biological consequences or overall genetic architecture. We investigated the prevalence and consequences of epistasis by analyzing four body composition phenotypes—body weight, body fat percentage, femoral density, and femoral circumference—in a large F2 intercross of B6-*lit/lit* and C3.B6-*lit/lit* mice. We used Combined Analysis of Pleiotropy and Epistasis (CAPE) to examine interactions for the four phenotypes simultaneously, which revealed an extensive directed network of genetic loci interacting with each other, circulating IGF1, and sex to influence these phenotypes. The majority of epistatic interactions had small effects relative to additive effects of individual loci, and tended to stabilize phenotypes towards the mean of the population rather than extremes. Interactive effects of two alleles inherited from one parental strain commonly resulted in phenotypes closer to the population mean than the additive effects from the two loci, and often much closer to the mean than either single-locus model. Alternatively, combinations of alleles inherited from different parent strains contribute to more extreme phenotypes not observed in either parental strain. This class of phenotype-stabilizing interactions has effects that are close to additive and are thus difficult to detect except in very large intercrosses. Nevertheless, we found these interactions to be useful in generating hypotheses for functional relationships between genetic loci. Our findings suggest that while epistasis is often weak and unlikely to account for a large proportion of heritable variance, even small-effect genetic interactions can facilitate hypotheses of underlying biology in well-powered studies.

## Author Summary

The role of statistical epistasis in the genetic architecture of complex traits has been of great interest to the genetics community since Fisher introduced the concept in 1918. However, assessing epistasis in human and model organism populations has been impeded by limited statistical power. To mitigate this limitation, we analyzed bone and body composition traits in an unusually large mouse intercross population of over 2000 mice, paired with a recently-developed computational approach that leverages information to detect interactions across multiple phenotypes. We discovered a large network of highly significant genetic interactions between variants that influence complex body composition traits. Although epistasis was abundant, the interaction network was dominated by epistasis that stabilizes phenotypes by reducing phenotypic deviation from the parent strains. Nevertheless, the observed network provides an overview of genetic architecture and specific hypotheses of how QTL combine to affect phenotypes. These findings suggest that epistatic effects are generally of lesser magnitude than main QTL effects, and therefore are unlikely to account for major components of variance, but also reinforce genetic interaction analysis as a potent tool for dissecting the biology of complex traits.

## Introduction

The relevance of epistasis in genetic architecture is yet unresolved. In genetic screens of model systems, the evidence for genetic interaction is abundant [1–4] and has been proven biologically relevant [5–7]. However, the situation is less clear in human populations as epistasis is difficult to detect with confidence due to multiple testing across a high number of variants, underpowered samples, evolutionary history, imperfect model selection, or unmeasured confounding variables or noise [8, 9]. While most studies detect only additive variance, recent studies have demonstrated a role of epistasis in the genetics of gene expression [10, 11] and occasionally link genetic interactions to disease [12]. Thus the extent to which genetic interactions contribute to unexplained variance or provide biological insight in population-based studies is unclear. One strategy to address this problem is controlled experiments in mammalian model systems, in which genotypes are artificially determined and environmental variation is minimized [13–16].

In this work, we used a multi-trait strategy to investigate the role of epistasis in regulating complex traits in a large mouse intercross. Bone mineral density (BMD) is a complex trait regulated by the interaction of many genetic and environmental factors [17–19], and is the best known surrogate measure of fracture risk in patients with osteoporosis [20–22]. Human and rodent studies have implicated many candidate quantitative trait loci (QTL) in influencing BMD [17–19, 23–27]. Many of these loci are pleiotropic and have been found to influence body weight [28], body fat [29–31], and bone size [32] in addition to bone density. Epistatic interactions are also common among loci affecting BMD [26, 33, 34]. A deep understanding of the genetic regulation of BMD, as well as possible intervention points for therapeutics, requires addressing this complex genetic architecture. Assessing genetic interactions and their relation to multiple phenotypes provides an overall picture of the genetic network regulating BMD and related phenotypes. We used a recently developed method, Combined Analysis of Pleiotropy and Epistasis (CAPE) [35], to integrate information across multiple phenotypes to infer directed genetic interactions between loci.

We integrated genetic interactions influencing BMD, femoral circumference, body weight, and body fat percentage in a mouse intercross population [36–39]. In particular, we were interested in investigating the genetic architecture of these phenotypes in a population with reduced levels of circulating insulin-like growth factor I (IGF1). IGF1 is a major factor involved in bone development and mineralization [40–43]. Analysis in a population with reduced IGF1 can reveal aspects of bone density that vary at severely reduced levels of IGF1 [44], thereby unmasking more subtle genetic loci involved in this phenotype [45, 46]. To avoid major effects of the IGF1/growth hormone (GH) axis we used mice homozygous for the “little” or *lit* mutation [47], a null mutation in the gene coding for growth hormone releasing hormone receptor (*Ghrhr*). GH levels, and consequently circulating IGF1 levels, in mice homozygous for the *lit* mutation are reduced to about 10% of wild type levels [37, 47, 48]. These mice also exhibit reduced growth, increased fat mass, and decreased bone mass relative to heterozygotes and wild type mice [49]. Thus *lit* homozygotes offer the opportunity to study the genetics related to both bone growth and body fat composition in a population in which one of the major hormonal axes regulating these phenotypes is greatly reduced.

A population of 2054 F_2_ male and female mice derived from a cross between B6-*lit/lit* and C3.B6-*lit/lit* parental strains [36–39] were analyzed. Compared to B6 mice, C3H mice have 20-30% higher circulating IGF1 levels, higher volumetric bone density, higher rates of bone formation, lower rates of bone resorption, and greater breaking strength of bones [50–52]. These strain differences persist in the *lit/lit* homozygotes [52]. We investigated the genetic interactions influencing body weight, percent body fat, femoral circumference and femoral density in the near absence of one of the major contributors to these phenotypes.

## Materials and Methods

### Ethics Statement

All animal procedures followed Association for Assessment and Accreditation of Laboratory Animal Care guidelines and were approved by Institutional Animal Care and Use Committee (The Jackson Laboratory, Protocol #99111).

### Mice

Inbred mouse strains used in this study were obtained from our research colonies at The Jackson Laboratory, Bar Harbor, Maine. Mice were produced and housed as described in [76]. Briefly, the mice were housed in same-sex groups of 2-5 per cage in a 14:10 light:dark cycle. The mice had free access to acidified water (pH 2.5 with HCl to retard bacterial growth) and irradiated NIH 31 diet (Purina Mills International, Brentwood, MO).

### Construction of Congenic Strain and F_2_ Intercross

To investigate heritable factors that control BMD in a model where circulating IGF1 levels are reduced, we used a spontaneous mouse mutation, *lit*, with a non-functional growth hormone releasing hormone receptor (*GHRHR*). We generated a congenic strain by transferring the *lit* mutation from the low-BMD C57BL/6J (B6) strain on which it arose to the high-BMD C3H/HeJ (C3H) strain by backcrossing for eighteen generations. In both C57BL/6J-*Ghrhr*^*lit/lit*^/J (B6-*lit/lit*) and C3H.B6-*Ghrhr*^*lit/lit*^/J (C3.B6-*lit/lit*) mice, circulating GH is undetectable, serum IGF1 is low, and femoral volumetric BMD by pQCT, femur length, and body mass are reduced compared to heterozygous *lit*/+ mice [37, 47, 48]. Although C3.B6-*lit/lit* mice are of the same body weight and femur length as B6-*lit/lit* mice, C3.B6-*lit/lit* mice have higher BMD. Crosses between B6-*lit/lit* and C3.B6-*lit/lit* F_1_ mice produced the 1008 male and 1062 female F_2_ GH/IGF1 deficient mice analyzed here.

### Genetic Analyses

Mice were genotyped at 100 markers using PCR of oligonucleotide primer pairs (MIT markers, www-genome.wi.mit.edu/cgi-bin/mouse/index) from Research Genetics (Birmingham, AL) as described in [76, 77]. The pairs amplified strain-specific sequence length polymorphisms, allowing identification of parental strain of origin. Genotypes at each locus were identified as B6/B6, B6/C3H, or C3H/C3H.

### Phenotype Measurements

#### Body weight and femur length

Anesthetized mice were weighed using a routinely calibrated Ohaus electronic scale. Femur length was measured using a digital caliper (Stoelting, Wood Dale, Ill).

#### Percent body fat by PIXImus

Data were collected on anesthetized mice using the PIXImus small animal DEXA system (LUNAR, Madison, WI), software version 1.43.036.008 as described in [78]. The machine was calibrated daily with a phantom of known density. Measurements of BMD showed low variability: less than 1% for whole body measurements, and about 1.5% for specialized regions. Body fat (BF) and percent body fat (%BF) were derived from measurements of total body weight (TBW), total lean mass (TLM), and total body mineral content (TBM) as follows: BF = TBW—(LBM + WBM); %BF = BF/TBW.

#### Femoral BMD and periosteal circumference by pQCT

The XCT 960M was used to measure total BMD at 2-mm intervals as described in [77] and [79]. Periosteal circumference was determined at midpoint of the total femur length. Precision of these measurements was determined to be 1.2% through repeated measurement of a single femur. Hydroxyapatite standards (0.050-1.000 mg/mm^3^) were used for calibration. The correlation between measured density and actual standard density was r = 0.997.

#### Serum IGF1

Serum IGF1 was measured by radioimmunoassay (RIA) (ALPCO, Windham, NH) as described in [80].

### Combined Analysis of Pleiotropy and Epistasis

CAPE is a strategy that detects epistasis and interprets it in terms of directed enhancing and suppressing influences between genetic loci [81]. It has been released as an R package suitable for mouse intercross studies [35]. The method uses regression on pairs of loci to detect interaction effects from each locus pair on each phenotype. It then combines model parameters across phenotypes to infer directed, QTL-to-QTL influences that replace the interaction effects on each phenotype. The result is a pair of directed coefficients modeling how the two loci influence each other’s activity, rather than how each pair independently affects each phenotype.

We began the analysis by using R/qtl [82] to impute psuedomarkers in between each measured marker, increasing the number of markers from 100 to 194. We normalized all traits (body weight, body fat percentage, femoral density, and femoral circumference) using rank Z normalization. We then decomposed the normalized traits using singular value decomposition (SVD) to obtain orthogonal eigentraits (ETs) that combined common signals across all phenotypes. All markers were used in the pair-wise interaction scans. However, we filtered marker pairs tested by linkage disequilibrium (LD) to avoid false positive interactions. We excluded all pairs with genotype Pearson correlation *r* ≤ 0.5. This reduced the number of pairs tested from 19,110 (all pairs from 194 markers and 2 covariates) to 18,576 pairs. Linear regression was then performed on all filtered pairs of markers 1 and 2:

$$\begin{array}{c} U_{i}^{j}=\beta_{0}^{j}+\underset{\text{covariates}}{\underset{︸}{\sum_{c=1}^{2}x_{c,i}\beta_{c}^{j}}}+\underset{\text{main}\;\text{effects}}{\underset{︸}{x_{1,i}\beta_{1}^{j}+x_{2,i}\beta_{2}^{j}}}+\underset{\text{interaction}}{\underset{︸}{x_{1,i}x_{2,i}\beta_{12}^{j}}}+\epsilon_{i}^{j} \end{array}$$

where *U* represents ETs, and *ϵ* is an error term. The index *i* runs from 1 to the number of individuals, and *j* runs from 1 to 3 (the number of ETs.) *x*_*i*_ is the probability of the presence of the C3H allele for individual *i* at locus *j*.

For each pair of markers, the regression coefficients are collected for all ETs and reparametrized to two new terms (*δ*_1_ and *δ*_2_). These terms represent the additional activity of each variant when the other is present. For example, *δ*_1_ is the additional effect that variant 1 has on each phenotype when variant 2 is present. It should be noted that the *δ* terms describe the interaction coefficient between the marker pair independent of phenotype. The *δ* are determined from the pairwise regression parameters via pseudoinversion as follows:

$$\begin{array}{c} \left[\begin{array}{c} \delta_{1}\\ \delta_{2} \end{array}\right]={\left[\begin{array}{cc} \beta_{1}^{1} & \beta_{2}^{1}\\ \beta_{1}^{2} & \beta_{2}^{2}\\ \beta_{1}^{3} & \beta_{2}^{3} \end{array}\right]}^{-1}\cdot \left[\begin{array}{c} \beta_{12}^{1}\\ \beta_{12}^{2}\\ \beta_{12}^{3} \end{array}\right] \end{array}$$

To convert the *δ* terms to directed influence variables, the following transformation was applied:

$$\begin{array}{c} \delta_{1}=m_{12}\left(1+\delta_{2}\right),\;\delta_{2}=m_{21}\left(1+\delta_{1}\right) \end{array}$$

The sign of the *m*_12_ and *m*_21_ terms indicates how each marker influences the other in terms of enhancement (positive) or suppression (negative). A negative value for *m*_21_ indicates that variant 1 reduces the activity of variant 2 on all phenotypes, whereas a positive value increases the activity. To estimate variances of the new model parameters, error estimates were propagated via second-order Taylor expansion [81, 83].

Permutation tests were used to calculate *p*-values for all model parameters. The pair of markers being tested was permuted together relative to the covariates [84]. By combining permutations across all marker pairs, we saved computation time while generating a single null distribution indistinguishable from the null distribution generated by repeatedly permuting each single pair. This null distribution was composed of 164,679 total permutations representing 3 permutations for each of 54,893 locus pairs.

Main effect significance was also determined through permutation testing. The maximum main effect of each locus across all pairwise contexts was selected as the main effect for that locus. All *p*-values were corrected for multiple testing using the Holm step-down procedure [85] and all variant-to-ET main effects were translated to variant-to-phenotype effects through multiplication by the singular value matrices from the original SVD.

### Grouping Linked Markers

To define distinct QTL regions for the interaction network, adjacent markers were combined into linkage blocks. We calculated the correlation matrix for all markers on each chromosome. We used this similarity matrix as an adjacency matrix to construct a weighted network depicting the similarity between all pairs of markers on a single chromosome. Using the fastgreedy community detection algorithm [86] in R/igraph [87], we then calculated the community membership of the vertices in the network. We assigned adjacent markers in the same community to a single QTL region. This process ensured a robust grouping of markers based on their genotypic similarity. A block was considered to have a significant effect on a phenotype if one or more of the resident markers had a significant effect. After this point, we refer to linkage blocks with significant associations as QTL or loci. See S3 Table for markers included in each block.

### Identification of Candidate Genes

Some of the groups of linked markers interacted significantly with IGF1, which is a sufficiently specific interaction to allow a candidate gene search. For each QTL that interacted significantly with IGF1 we generated a list of potential candidate genes by finding all genes in the QTL with annotations for bone density. To determine whether any of these genes interacted with IGF1, we used the Integrative Multi-species Prediction tool [88] to generate a network between the query genes and IGF1. We included the maximum of 50 additional genes and adjusted the confidence of the interactions until IGF1 was included in a subnetwork of greater than two genes. From this subnetwork, we extracted genes in the original QTL region, but which were not necessarily included the original query relating to bone density. Candidate polymorphisms in C3H alleles of these genes were identified using the Sanger SNP database [54]. Furthermore, we used previously published expression data from hepatic tissue of chow-fed B6 and C3H mice [56] to identify genes that were differentially expressed between the strains and pairs of genes that had correlated expression. Because most observed regulation of gene expression is in *cis*, it is reasonable to assume that the genetic variation that influences gene expression will influence expression levels similarly wherever the gene is expressed. Differential expression was determined with Student’s *t*-test, and significance of correlation was the significance of the Pearson correlation coefficient [89].

### Motif Analysis

We examined the enrichment of three-node topological patterns, or network motifs [58], in the set of all interactive genetic models. Although each interaction and main effect was independently derived, we grouped the significant effects into a network to detect general patterns. We combined interactions with main effects to generate three-node motifs, which included two interacting variants and a single phenotype. We counted enhancing and suppressing motifs either with two main effects of the same sign (coherent), or two main effects of the opposite sign (incoherent). To determine whether each type of motif was significantly enriched or depleted, we performed permutations by shuffling the signs of the significant interactions independently of the signs of the main effects. This permutation scheme preserved the topology of the network thereby acknowledging constraints caused by shared edges between motifs. By permuting the main effect edges independently of the interaction edges, we prevented spuriously enriching for enhancing or suppressing interactions simply because there were many negative or positive main effects on a given phenotype. We permuted the edge signs 100,000 times to generate a null distribution for each type of motif. The directions of the interactions were not taken into account for this analysis. We used linkage blocks as interacting units and included all interactions and main effects that were significant at a Holm-corrected *p* ≤ 0.01.

## Results

### Single-Locus Effects on Phenotypes

**Fig 1:**
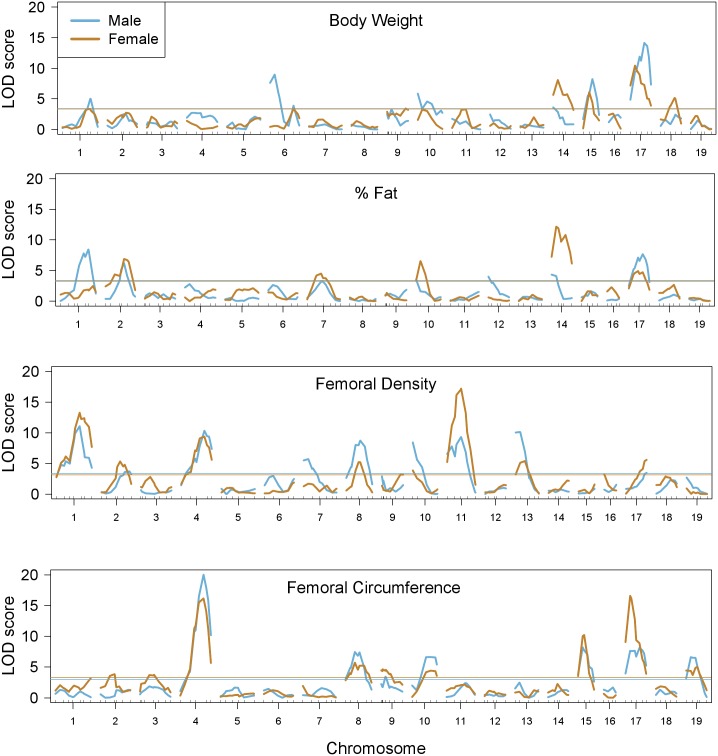
Single-locus associations with phenotypes. LOD scores for each locus and each phenotype for both males (blue) and females (brown). There are significant QTL for each of the phenotypes, and males and females tend to share QTL.

We used linear regression to determine the association of each locus with each phenotype (Fig 1, S4 Table). Each of the phenotypes had multiple associated QTL, and these QTL often overlapped across multiple phenotypes. For example, femoral density and femoral circumference shared a large QTL on Chr 4, and body weight, percent fat and femoral circumference showed overlapping QTL on Chr 17. These overlapping QTL indicate the possibility of common genetic factors underlying multiple phenotypes, such that information can be combined across multiple phenotypes to gain information about individual loci. Unique QTL were also observed, providing non-redundant information to discern genetic factors with phenotypic specificity.

### Single-Locus Effects on Eigentraits

**Fig 2:**
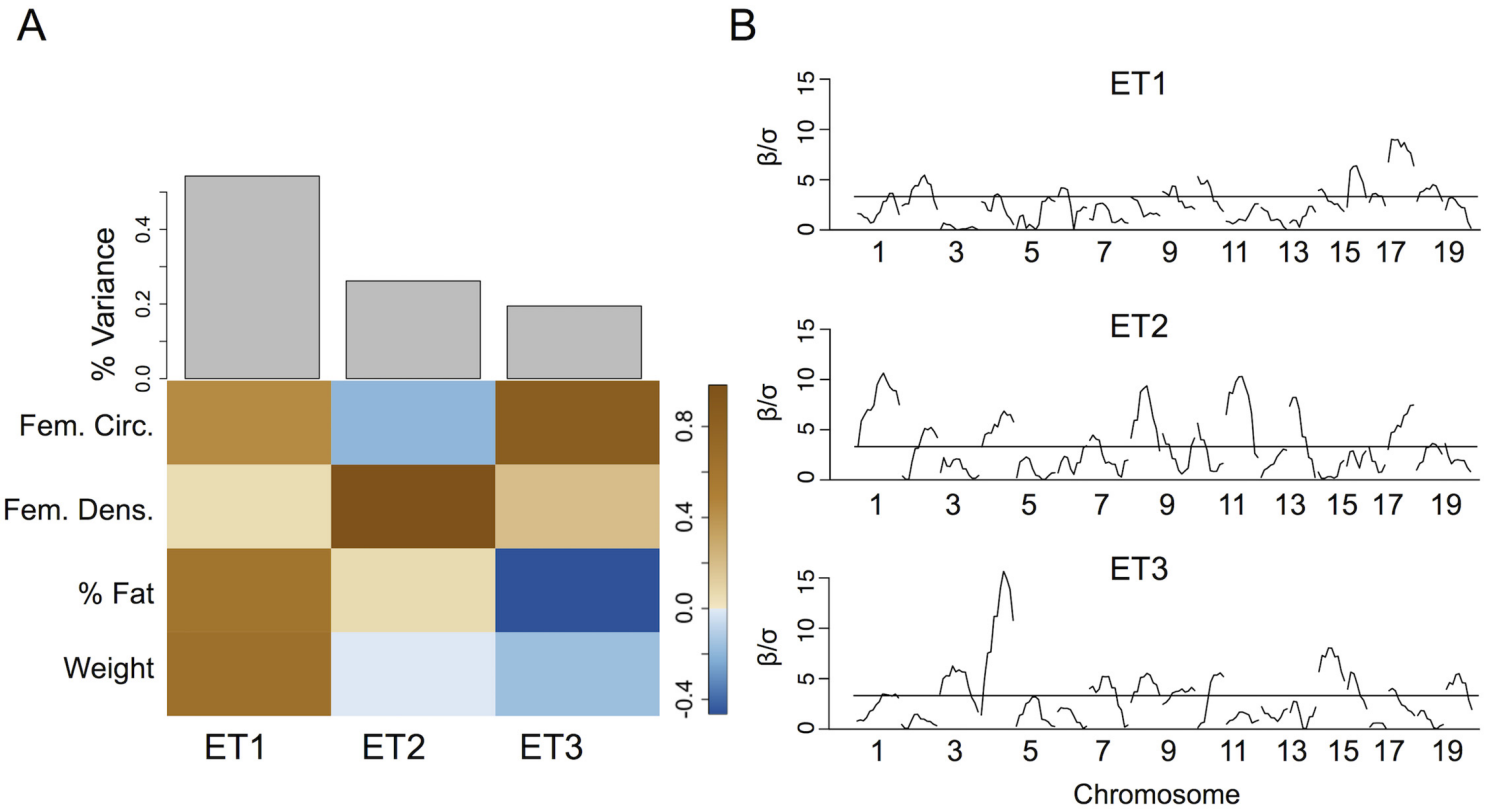
Single-locus associations with eigentraits. (A) Contributions of each phenotype to each eigentrait (ET). Brown squares indicate a positive contribution while blue squares indicate a negative contribution. Gray bars show the percent variance accounted for by each ET. (B) Standardized effects (*β*/*σ*) from the linear regression performed on each locus and each ET, with sex and IGF1 as covariates. There are significant QTL for each of the ET. Horizontal line represents the significance level at the permutation-based *p* = 0.05.

We decomposed the normalized, mean-centered phenotypes into eigentraits (ETs) (Fig 2A). The first ET represented an average of femoral circumference, percent body fat and body weight and captured 52% of the overall variance. The second ET represented the contrast between femoral circumference and femoral density and captured 25% of the total variance. The third ET captured 19% of the total variance and represented a contrast between femoral circumference and percent body fat. The fourth ET captured only 4% of the total variance, and described a contrast between body weight and percent fat. Because this ET captured a small amount of the total variance, did not include strong contributions from the bone phenotypes, and may add noise to the analysis, we excluded it from this analysis.

Single-locus associations with each ET detected multiple QTL (Fig 2B). Since ET are linear combinations of traits, each QTL indicates a potentially pleiotropic association with varying effect strengths on each trait. For example, data for body weight, percent fat and femoral circumference had overlapping QTL on Chr 17. These phenotypes also contributed substantially to ET1, and there was a corresponding significant QTL for ET1 on Chr 17 representing the common QTL.

### An Extensive Network of Weak Genetic Interactions Influences Bone and Body Composition

**Fig 3:**
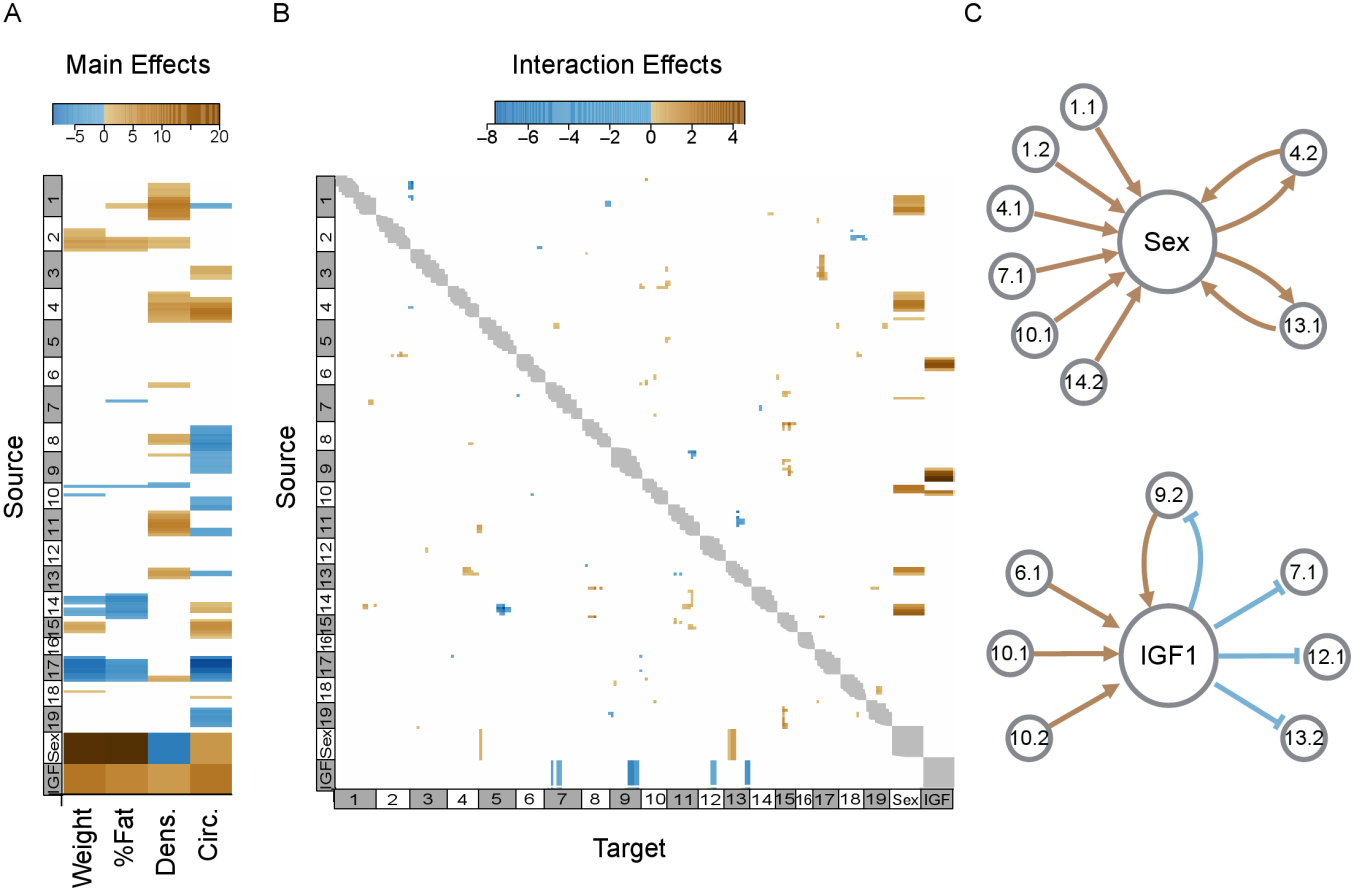
Main and interaction effects inferred by CAPE. (A) Standardized main effects (*β*/*σ*) of each marker on each phenotype. Only significant main effects (*p* ≤ 0.0005) are shown in color. Positive main effects are shown in brown and negative main effects are shown in blue. In this panel QTL main effects as well as main effects of sex and IGF1 are shown. (B) Standardized effect sizes (*m*/*σ*) of interactions between genetic markers and between genetic markers and sex and IGF1. Each interaction is directed and includes a source genetic marker and a target genetic marker. The interactions are read such that the row corresponds to the source marker and the column corresponds to the target marker. Significant interactions (*p* ≤ 0.0005) are shown in blue (suppressing) and brown (enhancing). Gray regions on the diagonal indicate marker pairs that were not tested due to linkage disequilibrium (*r* > 0.5). (C) Detailed depiction of interaction asymmetries between QTL (gray circles with QTL labels) and the two covariates, sex and IGF1, shown in (B). Brown lines indicate enhancing interactions and blue lines denote suppressing interactions.

CAPE analysis of the first three ETs produced a large network of genetic interactions (Fig 3). The high-confidence network (*p* ≤ 0.0005) consisted of a single connected component including 67 QTL linked by 102 directed interactions. Each interaction was directed from a *source locus* to a *target locus* and was either *suppressing* (negative), or *enhancing* (positive). In suppressing interactions the presence of the C3H allele at the source locus reduced the phenotypic effect of the C3H allele at the target locus regardless sign of the main effect. In enhancing interactions the presence of the C3H allele at the source locus increased the phenotypic effect of the C3H allele at the target locus regardless of the sign of the main effect. The QTL were distributed across the four phenotypes as follows: body weight had 13 QTL; percent fat had 11 QTL; femoral density had 19 QTL; and femoral circumference had 24 QTL. Among the interactions between QTL, 29 were suppressing and 73 were enhancing.

We also used standard linear regression to assess the effect of each marker pair interaction on each normalized phenotype. We calculated the pairwise interaction coefficients for all marker pairs including the two covariates, sex and circulating IGF1 levels. We calculated empirical *p*-values from 50,000 permutations and corrected the *p*-values for multiple testing using the Holm step-down procedure. No marker-marker or marker-covariate interactions were significant using the standard epistasis analysis after correction for multiple testing.

### Sex Interacts Significantly with Genetic Loci

Sex had significant main effects on all phenotypes. Males had substantially higher body weight (males: 22.0 g, females: 17.5 g; *p* ≤ 2 × 10^−16^) and percent body fat (males: 42%, females: 36%; *p* ≤ 2 × 10^−16^). Males also had slightly, but significantly higher femoral circumference (males: 3.81 mm, females: 3.78 mm; *p* = 2.9 × 10^−4^). Females had significantly higher BMD (males: 0.49 mg/mm^3^, females: 0.51 mg/mm^3^; *p* ≤ 2 × 10^−16^).

**Fig 4:**
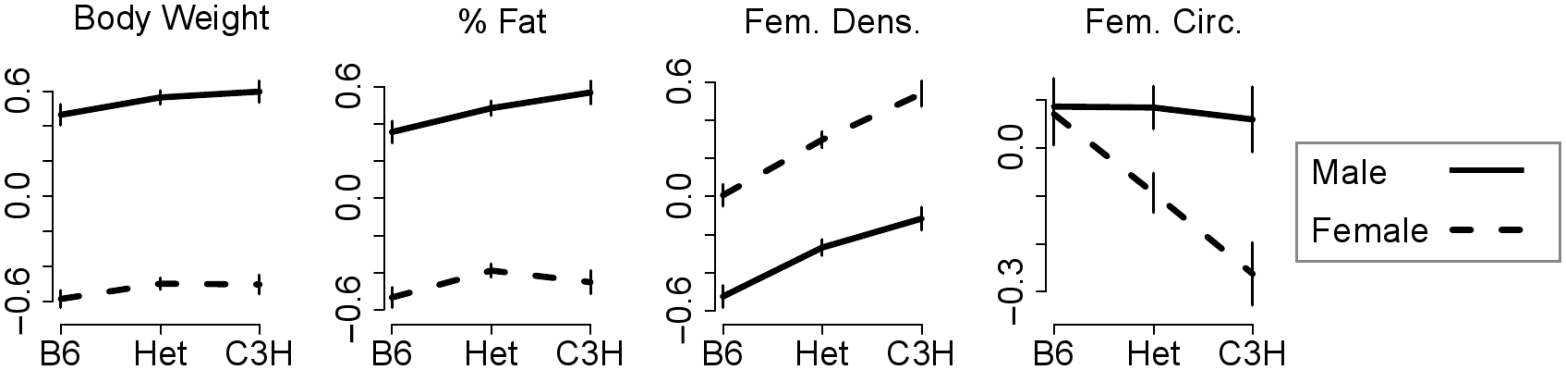
QTL interactions with sex. Phenotypes illustrating sex effects enhanced by a QTL on Chr 1. Males have higher body weight, body fat, and femoral circumference, and lower femoral density relative to females. The Chr 1 QTL clearly enhances these effects on femoral circumference, which is evident in the larger phenotypic effect of sex in mice homozygous for the C3H allele at the Chr 1 locus. There are smaller interaction effects for percent fat and femoral density. Importantly, the directed model for this interaction describes the results across all four phenotypes.

We found several significant sex-QTL interactions, all of which were enhancing (Fig 3C). From the single-locus regressions, potential sex-interacting loci were seen on Chrs 1, 6, 7, 10, and 14 (Fig 1). We tested these conditional associations directly and confirmed genetic interactions with sex on Chrs 1, 7, 10 and 14. For example, there was a larger sex difference in femoral density among animals homozygous for the C3H allele at the Chr 1 locus than among animals homozygous for the B6 allele (Fig 4). Thus the C3H status at this locus enhanced the negative effect of the male sex on femoral density, as well as the positive effect of the male sex on circumference, giving females with this genotype increased bone density and reduced circumference relative to males.

An apparent locus on Chr 6 (Fig 1) was not identified as interacting significantly with sex because a consistent directional model could not be fit across all phenotypes. Several markers on Chr 6 had relatively large interaction coefficients in the linear regression with sex, but none of these interactions were significant after correction for multiple testing.

### Residual IGF1 Interacts with Genetic Loci

**Fig 5:**
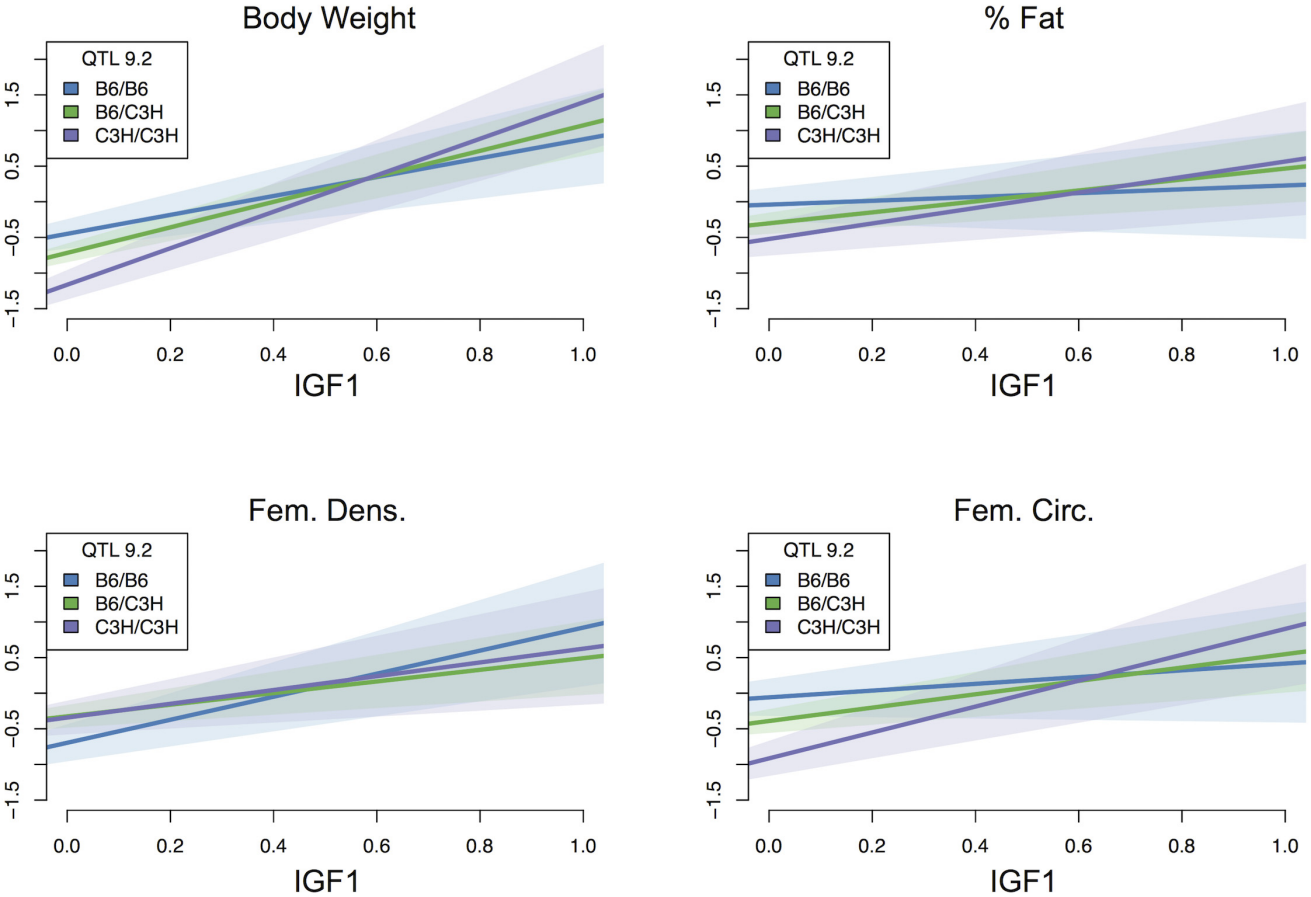
QTL interaction with IGF1. The effects of IGF1 are enhanced by QTL 9.2. IGF1 has a small positive effect on each phenotype in animals homozygous for the B6 allele at this locus. This can be seen in the positive slope of the blue line in each panel, which shows that each phenotype increases with increasing levels of IGF1. The effect of IGF1 on all phenotypes except femoral density is enhanced in heterozygotes (green line) and enhanced further in C3H homozygotes (purple line). For these genotypes, the increase in slope indicates that there is a more pronounced increase in phenotype as IGF1 levels increase. *Y*-axes represent rank normalized values of each phenotype, and *x*-axes represent rank normalized IGF1 levels scaled to range between 0 and 1. Shaded regions show 95% confidence interval for each slope.

Like sex, IGF1 had significant main effects on all phenotypes. Higher levels of circulating IGF1 were associated with higher body weight, body fat percentage, femoral density, and femoral circumference. IGF1 was also found to interact significantly with several QTL (Fig 3C). Interestingly, all interactions from IGF1 to loci were suppressing, *i.e*. IGF1 suppressed the effects of these loci. For example, one Chr 9 QTL had negative effects on both femoral density and femoral circumference when IGF1 levels were low, but at high levels of IGF1 this effect was suppressed, and the QTL had a positive effect on these phenotypes (Fig 5). Conversely, this locus also enhanced the effects of IGF1. Looking at body weight as a function of IGF1, for example, it can be seen that in the B6 homozygotes, IGF1 levels had a positive effect on body weight. There was a slightly larger effect in the heterozygotes, and the largest positive effect of IGF1 on body weight was in the C3H homozygotes (Fig 5). This general pattern is replicated across all phenotypes.

The molecular specificity of an interaction with IGF1 offered the opportunity to search for candidate genes in QTL interacting with IGF1, even though the regions were large (Methods). The second QTL on Chr 10 yielded promising candidates. Using MouseMine, we found 19 genes in the region with annotations to bone density [53]. We used these genes as a query gene set in the IMP tool. The IMP tool determines the likelihood that pairs of genes in a query set interact. It uses databases of known physical interactions, genetic interactions, and correlated expression to pull in additional genes though which genes in the query set may interact. The result is a network of high-confidence interactions that relate the query genes to each other. IMP found a network of 45 genes that linked IGF1 with four bone density-related genes (*Esr1*, *Nr1h4*, *Kitl*, *Ctgf*) from the query gene set. The minimum confidence for interactions between gene pairs in this network was 91%.

All four genes contain SNPs predicted to be functionally relevant between B6 and C3H [54], including splice site variants, missense variants and variants in regulatory regions (S5 Table). One of the genes, *Kitl* was differentially expressed in hepatic tissue between C3H and B6, with C3H mice showing lower expression (Student’s *t*-test, *p* = 0.012) [55, 56]. *Kitl* expression also showed a strong negative correlation with *Igf1* expression (*r* = −0.67, *p* = 0.016) [55, 56]. These findings allow us to hypothesize that *Kitl* is a potentially causative gene in QTL 10.2. Another potential candidate in this region, *Bicc1*, was recently found to be related to bone density in mice [57]. However, it was not differentially expressed in the hepatic tissue of C3H and B6 mice (Student’s *t*-test, *p* = 0.25) (S5 Table), and thus we consider it a less likely candidate in the context of this study. Other candidate regions did not reveal promising causative candidates when analyzed using the same methods.

### The Network Is Enriched for Stabilizing Motifs

**Fig 6:**
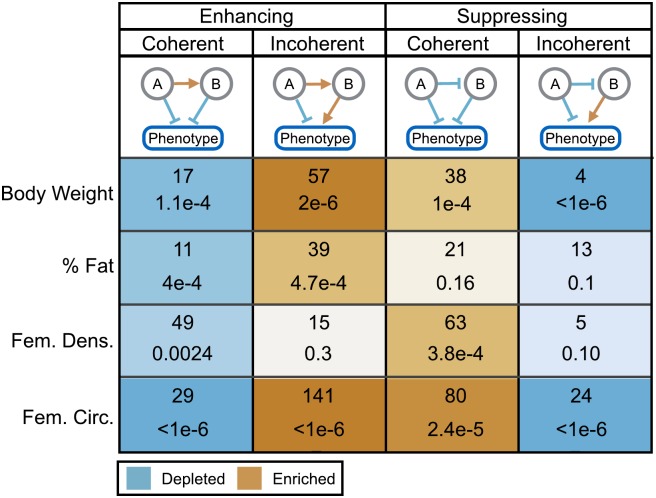
Enriched and depleted motifs in the CAPE genetic interaction network. The four possible motifs in the CAPE interaction network (*p* ≤ 0.01) are depicted in the top row. Variants A and B indicate the source and target QTL respectively in each motif type. Genetic interactions between QTL were either enhancing (positive) or suppressing (negative), and main effects were either coherent (same sign, positive or negative) or incoherent (opposite signs, one positive and one negative). Table cells show network motif count (top) and permutation-based enrichment *p*-value (bottom) for each motif type. Darker cells indicate greater significance of enrichment or depletion.

In addition to individual interactions, we examined the enrichment of three-node patterns, or network motifs [58] (Methods). Each motif consists of two interacting genetic loci and an affected phenotype. At significance *p* ≤ 0.01, we identified a total of 116 motifs influencing body weight, 84 motifs influencing percent fat, 132 motifs influencing femoral density, and 274 motifs influencing femoral circumference (Fig 6). Motifs are classified as coherent, *i.e*. the main effects are of the same sign, or incoherent, *i.e*. the main effects are of opposite signs. In addition, motifs can be either suppressing, meaning that there is a suppressing interaction between the genetic loci, or enhancing, with an enhancing interaction between the loci (Fig 6). These different classes of motifs may speak to the general structure of the underlying biological interactions [59–63]. For example, a motif with coherent main effects and a suppressing interaction between them indicates genetic redundancy and may result from proteins operating in series in the same pathway or physical interaction between gene products [60, 61]. Alternatively, a synergistic interaction exists in a coherent motif with an enhancing interaction between the variants. In this case, the variants and the interaction between them all push the phenotype in the same direction. Such an interaction may indicate regulatory interactions between parallel regulatory pathways that affect the same process [60].

These motifs can be divided into those that stabilize phenotypes and those that destabilize phenotypes. For example, incoherent enhancing motifs tended to have a stabilizing effect on phenotype because the main effects drove the phenotype in opposite directions. This was the largest class of motif represented in our network (42% of total motifs detected) and was significantly enriched across all phenotypes except femoral density (Fig 6). In contrast, coherent enhancing motifs tended to be destabilizing. The main effects of the interacting loci both drove the phenotype in the same direction, and the enhancing interaction between these loci drove the phenotype further in the same direction, generating extreme phenotypes. These enhancing coherent motifs made up only 17% of the total motifs detected and were significantly depleted across all phenotypes in our network (Fig 6).

Motifs with suppressing interactions were more evenly distributed. Coherent suppressing motifs, which tended to stabilize phenotypes, made up 33% of the total motifs and were enriched for all phenotypes except percent fat. Incoherent suppressing motifs, which tended to be destabilizing, were the smallest class of motif (8%) and were enriched in body weight and femoral circumference (Fig 6). In summary, the network overall consisted of weak interactions (mean *β* = 0.25±0.16) compared with the additive effects (mean *β* = 0.52±0.36), and the majority of the interactions (59%) were enhancing. Further, the two largest motif classes, making up 75% of the total motifs, tended to be stabilizing.

## Discussion

Bone density and body composition phenotypes, such as percent body fat, are complex traits influenced by many genetic variants, both shared and distinct. Here we used combined analysis of pleiotropy and epistasis (CAPE) to investigate how genetic loci interact in a large population of mice to influence femoral density, femoral circumference, body weight, and percent body fat. Using this exceptionally well-powered mouse intercross we detected many main-effect and interacting QTL associated with these traits and found an extensive network of genetic loci influencing the four phenotypes. These genetic interactions were not detectable through standard regression-based epistasis analysis. We were also able to infer both the directionality and sign of the interactions, which improved our ability to identify candidate QTL genes and provided a uniquely broad view of the genetic architecture of bone and body composition phenotypes. One of the notable features of the network was an asymmetric distribution of enhancing and suppressing interactions, which was apparent for interactions between QTL, as well as for QTL interactions with sex and circulating IGF1.

Sex showed a strong bias in both the directionality and sign of interactions (Fig 3B). Most (8 of 10) interactions were QTL that enhanced the phenotypic effects of the male sex on each phenotype. For example, the presence of the C3H alleles in QTL 1.1 increased the positive effect that the male sex had on weight, percent fat, and femoral circumference, as well as the negative effect the male sex had on femoral density. The remaining interactions (2 of 10) showed effects in the opposite direction. For example, being male enhanced the positive main effect that QTL 4.2 had on femoral circumference and density (Fig 3C). Sex and sex hormones are known to influence bone growth and density [64–66] and genetic loci that interact with sex to influence bone phenotypes have also been previously identified in rodent models [23, 27, 33, 67, 68]. That the QTL were the sources and sex was the target of the majority of these interactions highlights how CAPE determines directionality and interpretation of interactions. Sex was widely pleiotropic, affecting all phenotypes significantly (Fig 3A). An interaction in which sex is a target implies that the QTL influences all of these phenotypes to be identified by CAPE via its modifications on sex. Conversely, sex can target a non-pleiotropic QTL to influence individual phenotypes or a subset of phenotypes. It is possible that the sex-enhancing QTL contain variants in endocrine genes that globally affect sex effects. The two sex-enhanced QTL (4.2 and 13.1), which reciprocally enhance sex effects, are loci for which the uniform enhancement of the sex effects was insufficient to fit all phenotypes simultaneously. These QTL are therefore more likely to be involved in processes that differentially affect the phenotypes, suggesting more specific roles in each phenotype that are responsive to sex difference. These findings imply that interactions between sex and QTL are commonly due to genes that lie “upstream” of processes that underlie sexual dimorphism, rather than “downstream” genes with functions that are altered by sex hormones.

Circulating IGF1, which is reduced to 10% of wild type levels in this *lit/lit* population, had both main effects and interaction effects. It suppressed the effects of four loci, whereas four loci enhanced the effects of circulating IGF1 (Fig 3C). In contrast to sex, the interactions that IGF1 participates in are relatively balanced between incoming and outgoing interactions. This balance may reflect the fact that, unlike sex, IGF1 is a specific protein that physically interacts with other proteins. We interpret the role of the loci suppressed by IGF1 to be compensatory pathways influencing bone density when IGF1 levels are extremely low. At higher levels of circulating IGF1 the effects of these loci are diminished because the role of the causal genes becomes less relevant. For example, QTL 7.1 has a positive main effect on femoral density, which is suppressed by the presence of density-promoting IGF1. Conversely, the loci that enhance the effects of IGF1 may be targets of IGF1 that act to increase bone density and other phenotypes when IGF1 is present. These QTL, *e.g*. QTL 10.2 discussed below, can therefore be interpreted to contain genes in pathways that regulate and/or respond to IGF1 signaling. We note that one QTL, Chr 9.2, acts as an enhancer of IGF1 and is suppressed by IGF1 (Fig 3C). This QTL may therefore contain multiple genes involved in both IGF1 pathways and compensatory pathways, or be the result of a gene with an IGF1 signaling role that also serves to trigger alternative pathways in the absence of circulating IGF1.

**Fig 7:**
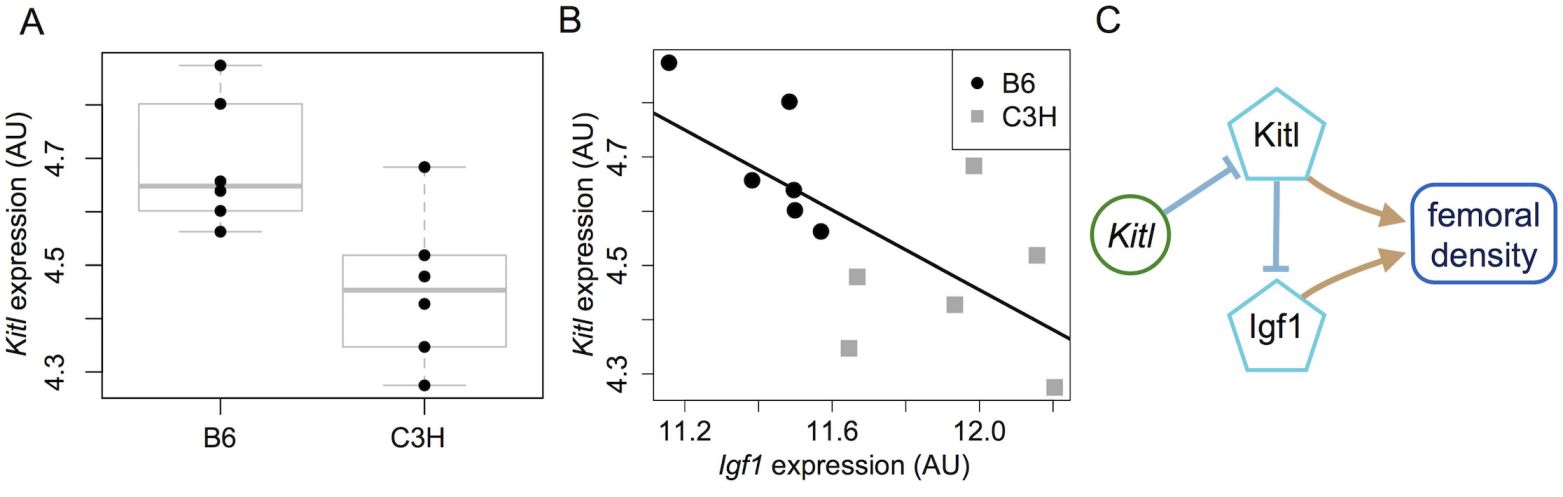
*Kitl* as a negative regulator of IGF1 models how QTL 10.2 enhances IGF1 effect on femoral density. Kitl transcript expression is (A) significantly lower in C3H mice than in B6 mice (*p* = 0.012), and (B) negatively correlated with *Igf1* expression (*r* = −0.67, *p* = 0.018) [55, 56]. (C) Model of the relationships between the *Kitl* C3H allele (green circle), the *Kitl* and *Igf1* transcripts (teal pentagons), and femoral density. The C3H allele of *Kitl* decreases the gene’s expression resulting in the QTL 10.2 negative effect on femoral density. Independently, the *lit* mutation reduces the levels of *Igf1* transcript and circulating IGF1. Residual *Igf1* transcript is negatively regulated by the *Kitl* transcript in B6 mice, but the reduced *Kitl* transcript levels in C3H mice permit increased IGF1 effects on femoral density. This yields an incoherent enhancing genetic interaction from QTL 10.2 to IGF1 (Fig 3C).

Although our QTL intervals were too large to decisively identify positional candidate genes, results for main effects and genetic interactions can be combined with prior data to reason about potential candidates. As an example, our model for QTL 10.2 interaction with IGF1 illustrates how hypotheses of causal QTL genes can be generated by requiring consistency in both main effects and interactions. Of the genes in QTL 10.2, *Kitl* had the best evidence to suggest a role in interacting with IGF1 to influence bone density. In our study we found that the C3H allele at QTL 10.2 had a negative main effect on femoral density. Prior work determined that hepatic *Kitl* expression is lower in C3H mice than in B6 mice (Student’s *t*-test, *p* = 0.012) [56] (Fig 7A) and that low expression is potentially associated with low femoral density [57]. Taken together these data suggest that the C3H allele of *Kitl* may be a loss of function variant that reduces *Kitl* transcript levels and consequently femoral density. Furthermore, this hypothesis can account for the directional interaction between QTL 10.2 and IGF1, in which the C3H allele at QTL 10.2 enhanced the positive effects of IGF1 on femoral density. The hepatic expression study found that *Kitl* and *Igf1* are negatively correlated (*r* = −0.67, *p* = 0.016) (Fig 7) [56] (Fig 7B). We can therefore hypothesize that a decrease in *Kitl* expression from the C3H allele corresponds to a rise in IGF1 activity and consequently its positive effects on femoral density. Combining the evidence for an interaction between *Kitl* and circulating IGF1 with the main effects of *Kitl* and QTL 10.2 suggests that increased *Kitl* transcript acts to reduce IGF1 activity in the reference B6 genotype (Fig 7C). When the C3H allele is present *Kitl* expression decreases, allowing residual *Igf1* transcript levels to remain relatively high and thereby enhancing the effect of IGF1 on femoral density in C3H mice relative to B6 mice. While speculative, this capacity to generate specific molecular hypotheses by combining genetic interactions with prior molecular results illustrates the importance of genetic interactions in hypothesis generation, even when they are a minor correction to additive effects. Such analysis is expected to be especially effective in a study with greater genetic mapping resolution and fewer candidate genes per QTL.

Our interaction network was derived in a population homozygous for the *lit* mutation, a receptor variant that perturbs IGF1 levels. One possible consequence is that the measured phenotypes have been decanalized and some of the QTL we observed may correspond to cryptic genetic variation [45, 46, 69] that only influence traits in the presence of the *lit/lit* mutation. This release of cryptic variation may be due to IGF1 effects being reduced to the point that minor genetic effects become observable. Alternatively, low levels of IGF1 induce activity in pathways that are not used in non-*lit* mice for maintaining growth and bone density, thereby making variation in the genes in these pathways relevant [46]. Potentially cryptic variants could be identified as those which do not replicate in a similar analysis of standard B6 and C3H strains, although resolution of compensatory pathways would likely require a conditional *Igf1* knockout.

**Fig 8:**
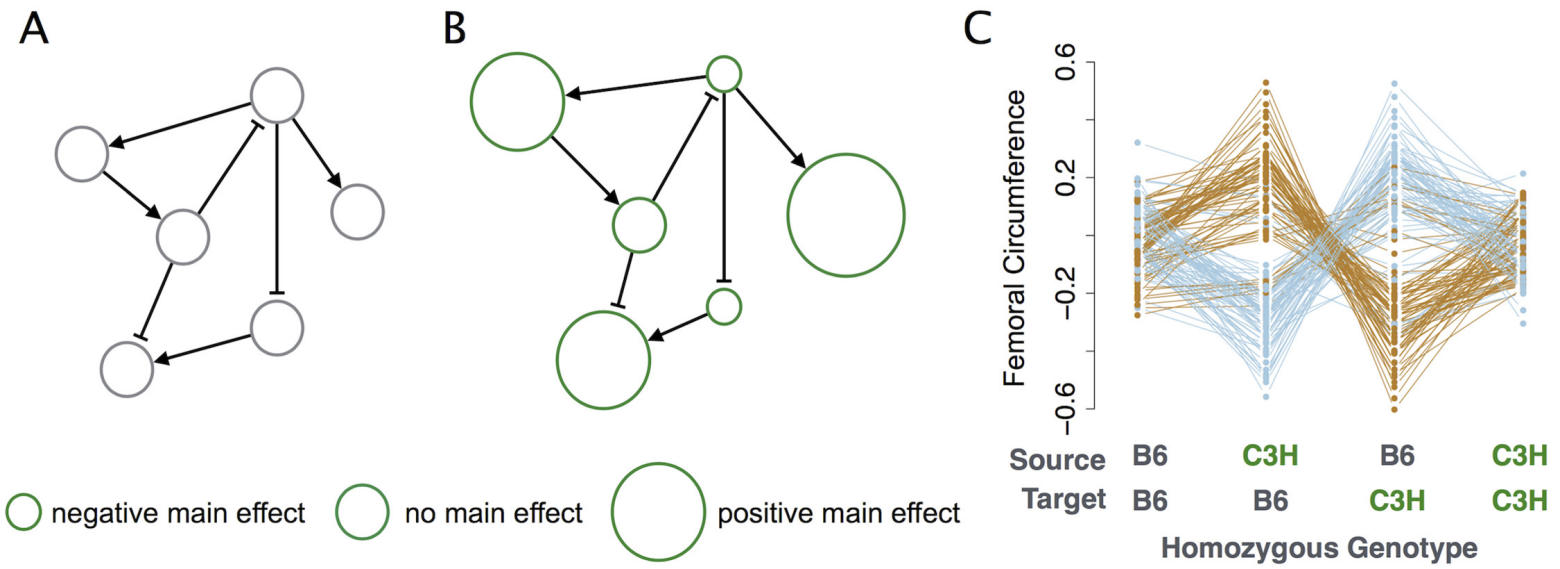
Enhancing incoherent motifs reduce extreme phenotypes. (A) Hypothetical gene regulatory network in a B6 mouse. All alleles have the reference main effect and interact to generate the reference phenotype. (B) The same regulatory network in a C3H mouse. Relative to the B6 alleles, the C3H alleles have negative, positive, or neutral effects. The C3H alleles combine additively and interactively to balance main effects and yield a phenotype similar to that of the B6 mouse. Taken in pairs, the most common outcomes will be: (i) balanced opposite-effect alleles that additively mimic the reference phenotype; (ii) imbalanced opposite-effect alleles that approach the reference phenotype, thereby revealing an incoherent enhancing interaction; or (iii) same-effect alleles will redundantly affect the phenotype either positively or negatively, thereby exhibiting a coherent suppressing interaction. Synergistic interactions (coherent enhancing or incoherent suppressing) are relatively rare in this model. (C) These network properties are manifest as pair-wise genetic interactions that reduce phenotypic variance when both loci share ancestry, as shown for femoral circumference. Mean phenotypes are plotted by genotype for each incoherent enhancing interaction (*N* = 141). Genotype refers to the homozygous genotype at each locus, either the source locus or the target locus. Heterozygous animals, which display intermediate phenotypes, are excluded for clarity. Source and target loci are defined by CAPE directional analysis. Interactions with a negative-effect source variant and positive-effect target variant are plotted in blue, with the opposite configuration in brown. Double-homozygote animals exhibit a reduced range of phenotype values than those with different alleles at the two loci, which have a broader range of phenotypes.

Overall, the QTL-QTL interaction network in this study had significant enrichment of enhancing interactions (Fig 6). Rather than instances of classic genetic synergy in which two variants combine to amplify a common effect, these enhancing interactions were mostly between variants with incoherent (opposing) main effects. We interpret these interactions as a signature of similar phenotypes between B6 and C3H strains that arise from different combinations of alleles. When alleles from two different strains are recombined in novel ways, unanticipated variation is introduced and extreme phenotypes result (Fig 8). Thus our enhancing interactions indicate a reduction of extreme phenotypes when both loci are homozygous for either parental allele. This moderating effect only occurs if the interaction between incoherent variants is enhancing. For any given phenotype an enhancing interaction from a positive main effect to a negative main effect is equivalent to a suppressing interaction in the reverse direction (Fig 6). However, when this model is applied across multiple phenotypes it is more likely to lead to high phenotype variability in the double homozygotes. This is because the suppressing QTL reduces an opposing main effect thereby favoring its own main effect. In contrast, in the enhancing model the enhancing QTL increases the opposing effect, thus balancing the effect of the overall interaction and bringing the phenotype toward the parental mean.

Although additive genetic variance is usually sufficient to equalize opposing QTL effects, when the positive and negative main effects are of unequal magnitude, genetic interactions are required to stabilize the phenotypes at the levels seen in the founders. Our network outcomes suggest that genetic interactions in intercross populations will commonly have relatively weak effects, since they are a fractional correction to main effects. This can be contrasted with extreme phenotypes that arise from synergistic (coherent enhancing) interactions in which interaction effects are of greater magnitude than main effects, such as in the classic case of genetic buffering [70]. These interactions are significantly depleted in our network.

Enrichment for motifs that drive phenotypes toward founder values is consistent with previous observations of phenotypic variation in recombinant populations. In populations of mice and *Drosophila* with introgressed genomic regions, simple additive contributions from all variants would result in phenotypic variance orders of magnitude greater than is observed between founders. That the founders have reduced phenotypic variance between them suggests that most interactions are less than additive [71–74]. Here, we see an enrichment of motifs leading to a reduction of extreme phenotypes, namely, suppressing coherent motifs, and enhancing incoherent motifs. While these effects are often weaker than main effects and therefore may not substantially improve the heritability accounted for, they nevertheless indicate genes acting together in a common pathway or process [60, 61]

Our analysis of a large intercross population has revealed a number of features that may be generalized to the genetic architecture of complex traits. First, we have found that a sufficiently powered study paired with a multi-trait analysis method can reveal a large network of genetic interactions between QTL. The systematic patterns in this network, including interactions with sex and a molecular marker, suggest that the interactions are signatures of the pathways and processes involved in the regulation of complex physiological traits. Second, we have found that the most common type of genetic interaction is a fairly subtle signal arising from allelic combinations that drive phenotypes towards median rather than extreme values. These interactions are either minor deviations from additivity or involve alleles with redundant effects, with the former being particularly difficult to detect in all but the largest study populations. These findings are consistent with recent work on a very large meta-analysis of twin studies [75]. Third, we note that the genetic interactions detected here form a connected network involving many interactions between the same subsets of QTL. We speculate that this is because the casual variants reside in groups of co-functional genes that compose specific pathways or processes, and that these pathways vary at multiple points between the B6 and C3H inbred strains. Since pair-wise combinations of C3H alleles have been shown to interactively drive the phenotypes towards the median, we speculate that as more genomic regions from C3H are inherited in a single individual, higher-order combinations of C3H alleles within these pathways will further canalize toward the C3H phenotype rather than cause large phenotypic variation. This may contribute to higher-order epistasis, since many of the variants will have strong effects in isolation that vanish in combinations. In sum, these findings suggest that the most common forms of epistasis may often be difficult to detect, and that the analysis of genetic interactions is nevertheless a powerful means to understand the genetics of the underlying biological pathways and processes.

## Supporting Information

S1 Table Data used in this analysis formatted for CAPE. A comma-separated file containing phenotypes and genotypes used in this analysis. The first six columns contain covariates (sex and IGF1) as well as the phenotypes. The remaining columns contain the name, chromosome, position, and genotypes for 100 MIT markers.
(CSV)

S2 Table A comma-separated file containing information for all tested pairwise interactions and main effects. The table is sorted by decreasing effect size, and each row contains the names of the source and target markers (or maker and phenotype for main effects), the effect size of the interaction (or main effect), the standard error, the standardized effect size, the empirical *p*-value as calculated from permutations, and the Holm-corrected *p*-value.
(CSV)

S3 Table A tab-delimited file containing the markers assigned to each linkage block. The first column displays the name of the linkage block, and subsequent columns show the names of the markers in the linkage block. All marker names are prefixed with a chromosome label. Markers designated “locX” are imputed pseudomarkers obtained from R/qtl (Methods).
(TXT)

S4 Table Single-Marker Scan Information by Linkage Block. A tab-delimited file containing information about linkage block effects in the single-marker scan. Information includes the number of markers in each linkage block, genomic start and stop positions, standardized effect sizes and significance levels for each eigentrait, as well as a gene count for the block. Only protein coding genes were included in the count.
(TXT)

S5 Table Candidate Genes. A tab-delimited file containing information about genes that are likely candidates for interacting with IGF1 to influence bone density. The table lists evidence to support each gene’s candidacy.
(TXT)

## References

[1] Tong AHY, Evangelista M, Parsons AB, Xu H, Bader GD, et al (2001) Systematic genetic analysis with ordered arrays of yeast deletion mutants. Science 294: 2364–2368. 10.1126/science.1065810 11743205

[2] Drees BL, Thorsson V, Carter GW, Rives AW, Raymond MZ, et al (2005) Derivation of genetic interaction networks from quantitative phenotype data Genome Biology 6: R38. 15833125 10.1186/gb-2005-6-4-r38 PMC1088966

[3] Huang W, Richards S, Carbone MA, Zhu D, Anholt RRH, et al (2012) Epistasis dominates the genetic architecture of drosophila quantitative traits. Proceedings of the National Academy of Sciences 109: 15553–15559. 10.1073/pnas.1213423109 PMC3465439 22949659

[4] Lehner B, Crombie C, Tischler J, Fortunato A, G FA (2006) Systematic mapping of genetic interactions in Caenorhabditis elegans identifies common modifiers of diverse signaling pathways. Nature Genetics 38: 896–903. 10.1038/ng1844 16845399

[5] Costanzo M, Baryshnikova A, Bellay J, Kim Y, Spear ED, et al (2010) The genetic landscape of a cell. Science 327: 425–431. 10.1126/science.1180823 20093466 PMC5600254

[6] Carter GW, Prinz S, Neou C, Shelby JP, Marzolf B, et al (2007) Prediction of phenotype and gene expression for combinations of mutations. Molecular Systems Biology 3: 96 10.1038/msb4100137 17389876 PMC1847951

[7] Collins SR, Miller KM, Maas NL, Roguev A, Fillingham J, et al (2007) Functional dissection of protein complexes involved in yeast chromosome biology using a genetic interaction map. Nature 446: 806–810. 10.1038/nature05649 17314980

[8] Mackay TF, Moore JH (2014) Why epistasis is important for tackling complex human disease genetics. Genome Medicine 6:42. 25031624 10.1186/gm561 PMC4062066

[9] Wei WH, Hemani G, Haley CS (2014) Detecting epistasis in human complex traits. Nature Reviews Genetics 15: 722–733. 10.1038/nrg3747 25200660

[10] Powell JE, Henders AK, McRae AF, Kim J, Hemani G, et al (2013) Congruence of additive and non-additive effects on gene expression estimated from pedigree and snp data. PLoS Genetics 9: e1003502 10.1371/journal.pgen.1003502 23696747 PMC3656157

[11] Hemani G, Shakhbazov K, Westra HJ, Esko T, Henders AK, et al (2014) Detection and replication of epistasis influencing transcription in humans. Nature 508: 249–253. 10.1038/nature13005 24572353 PMC3984375

[12] Westra HJ, Peters MJ, Esko T, Yaghootkar H, Schurmann C, et al (2013) Systematic identification of trans eqtls as putative drivers of known disease associations. Nature Genetics 45: 1238–1243. 10.1038/ng.2756 24013639 PMC3991562

[13] Churchill GA, Airey DC, Allayee H, Angel JM, Attie AD, et al (2004) The collaborative cross, a community resource for the genetic analysis of complex traits. Nature Genetics 36: 1133–1137. 10.1038/ng1104-1133 15514660

[14] Valdar W, Solberg LC, Gauguier D, Burnett S, Klenerman P, et al (2006) Genome-wide genetic association of complex traits in heterogeneous stock mice. Nature Genetics 38: 879–887. 10.1038/ng1840 16832355

[15] Svenson KL, Gatti DM, Valdar W, Welsh CE, Cheng R, et al (2012) High-resolution genetic mapping using the mouse diversity outbred population. Genetics 190: 437–447. 10.1534/genetics.111.132597 22345611 PMC3276626

[16] Buchner DA, Burrage LC, Hill AE, Yazbek SN, O’Brien WE, et al (2008) Resistance to diet-induced obesity in mice with a single substituted chromosome. Physiological Genomics 35: 116–122. 10.1152/physiolgenomics.00033.2008 18628339 PMC2536825

[17] Ralston SH, de Crombrugghe B (2006) Genetic regulation of bone mass and susceptibility to osteoporosis. Genes & Development 20: 2492–2506. 10.1101/gad.1449506 16980579

[18] Raisz LG (2005) Pathogenesis of osteoporosis: concepts, conflicts, and prospects. The Journal of Clinical Investigation 115: 3318–3325. 10.1172/JCI27071 16322775 PMC1297264

[19] Mitchell BD, Streeten EA (2013) Clinical impact of recent genetic discoveries in osteoporosis. The Application of Clinical Genetics 6: 75–85. 10.2147/TACG.S52047 24133373 PMC3796859

[20] Kanis JA, Melton LJ, Christiansen C, Johnston CC, Khaltaev N (1994) The diagnosis of osteoporosis. Journal of Bone and Mineral Research 9: 1137–1141. 10.1002/jbmr.5650090802 7976495

[21] Lips P (1997) Epidemiology and predictors of fractures associated with osteoporosis. The American Journal of Medicine 103: 3S–8S. 10.1016/S0002-9343(97)90021-8 9302892

[22] Siris ES, Miller PD, Barrett-Connor E, Faulkner KG, Wehren LE, et al (2001) Identification and fracture outcomes of undiagnosed low bone mineral density in postmenopausal women: results from the National Osteoporosis Risk Assessment. JAMA 286: 2815–2822. 10.1001/jama.286.22.2815 11735756

[23] Beamer WG, Shultz KL, Ackert-Bicknell CL, Horton LG, Delahunty KM, et al (2007) Genetic dissection of mouse distal chromosome 1 reveals three linked BMD QTLs with sex-dependent regulation of bone phenotypes. Journal of Bone and Mineral Research 22: 1187–1196. 10.1359/jbmr.070419 17451375

[24] Farber CR, Bennett BJ, Orozco L, Zou W, Lira A, et al (2011) Mouse Genome-Wide Association and Systems Genetics Identify Asxl2 As a Regulator of Bone Mineral Density and Osteoclastogenesis. PLoS Genetics 7: e1002038 10.1371/journal.pgen.1002038 21490954 PMC3072371

[25] Ishimori N, Li R, Walsh KA, Korstanje R, Rollins JA, et al (2006) Quantitative trait loci that determine BMD in C57BL/6J and 129S1/SvImJ inbred mice. Journal of Bone and Mineral Research 21: 105–112. 10.1359/JBMR.050902 16355279

[26] Zhang F, Xiao P, Yang F, Shen H, Xiong DH, et al (2008) A whole genome linkage scan for QTLs underlying peak bone mineral density. Osteoporosis International 19: 303–310. 10.1007/s00198-007-0468-z 17882466

[27] Koller DL, Liu L, Alam I, Sun Q, Econs MJ, et al (2008) Linkage screen for BMD phenotypes in male and female COP and DA rat strains. Journal of Bone and Mineral Research 23: 1382–1388. 10.1359/JBMR.080401 18707222 PMC2683154

[28] Klein RF, Mitchell SR, Phillips TJ, Belknap JK, Orwoll ES (1998) Quantitative trait loci affecting peak bone mineral density in mice. Journal of Bone and Mineral Research 13: 1648–1656. 10.1359/jbmr.1998.13.11.1648 9797472

[29] Rosen CJ, Ackert-Bicknell CL, Adamo ML, Shultz KL, Rubin J, et al (2004) Congenic mice with low serum IGF-I have increased body fat, reduced bone mineral density, and an altered osteoblast differentiation program. Bone 35: 1046–1058. 10.1016/j.bone.2004.07.008 15542029

[30] Mehrabian M, Allayee H, Stockton J, Lum PY, Drake TA, et al (2005) Integrating genotypic and expression data in a segregating mouse population to identify 5-lipoxygenase as a susceptibility gene for obesity and bone traits. Nature Genetics 37: 1224–1233. 10.1038/ng1619 16200066

[31] Steppan CM, Crawford DT, Chidsey-Frink KL, Ke H, Swick AG (2000) Leptin is a potent stimulator of bone growth in ob/ob mice. Regulatory Peptides 92: 73–78. 10.1016/S0167-0115(00)00152-X 11024568

[32] Wang X, Kammerer CM, Wheeler VW, Patrick AL, Bunker CH, et al (2007) Pleiotropy and heterogeneity in the expression of bone strength-related phenotypes in extended pedigrees. Journal of Bone and Mineral Research 22: 1766–1772. 10.1359/jbmr.070718 17931101

[33] Koller DL, Liu L, Alam I, Sun Q, Econs MJ, et al (2008) Epistatic effects contribute to variation in BMD in Fischer 344 x Lewis F2 rats. Journal of Bone and Mineral Research 23: 41–47. 10.1359/JBMR.071001 17907919 PMC2663590

[34] Yang TL, Shen H, Xiong DH, Xiao P, Guo Y, et al (2007) Epistatic interactions between genomic regions containing the COL1A1 gene and genes regulating osteoclast differentiation may influence femoral neck bone mineral density. Annals of Human Genetics 71: 152–159. 10.1111/j.1469-1809.2006.00313.x 17331078

[35] Tyler AL, Lu W, Hendrick JJ, Philip VM, Carter GW (2013) CAPE: An R Package for Combined Analysis of Pleiotropy and Epistasis. PLoS Computational Biology 9: e1003270 10.1371/journal.pcbi.1003270 24204223 PMC3808451

[36] Kohler T, Stauber M, Donahue LR, Müller R (2007) Automated compartmental analysis for high-throughput skeletal phenotyping in femora of genetic mouse models. Bone 41: 659–667. 10.1016/j.bone.2007.05.018 17662679

[37] Mohan S, Richman C, Guo R, Amaar Y, Donahue LR, et al (2003) Insulin-like growth factor regulates peak bone mineral density in mice by both growth hormone-dependent and -independent mechanisms. Endocrinology 144: 929–936. 10.1210/en.2002-220948 12586770 PMC2923925

[38] Ruffoni D, Kohler T, Voide R, Wirth AJ, Donahue LR, et al (2013) High-throughput quantification of the mechanical competence of murine femora–a highly automated approach for large-scale genetic studies. Bone 55: 216–221. 10.1016/j.bone.2013.02.015 23486181

[39] Schneider P, Stauber M, Voide R, Stampanoni M, Donahue LR, et al (2007) Ultrastructural properties in cortical bone vary greatly in two inbred strains of mice as assessed by synchrotron light based micro- and nano-CT. Journal of Bone and Mineral Research 22: 1557–1570. 10.1359/jbmr.070703 17605631

[40] Elis S, Courtland HW, Wu Y, Rosen CJ, Sun H, et al (2010) Elevated serum levels of IGF1-1 are sufficient to establish normal body size and skeletal properties even in the absence of tissue IGF-1. Journal of Bone and Mineral Research 25: 1257–1266. 10.1002/jbmr.20 20200935 PMC3153133

[41] He J, Rosen CJ, Adams DJ, Kream BE (2006) Postnatal growth and bone mass in mice with IGF-I haploinsufficiency. Bone 38: 826–835. 10.1016/j.bone.2005.11.021 16427371

[42] Sjögren K, Sheng M, Movérare S, Liu JL, Wallenius K, et al (2002) Effects of liver-derived insulin-like growth factor I on bone metabolism in mice. Journal of Bone and Mineral Research 17: 1977–1987. 10.1359/jbmr.2002.17.11.1977 12412805

[43] Martari M, Salvatori R (2009) Diseases associated with growth hormone-releasing hormone receptor (GHRHR) mutations. Progress in Molecular Biology and Translational Science 88: 57–84. 10.1016/S1877-1173(09)88003-4 20374725

[44] Donahue L, Guido V, Rosen C, Horton L, Ackert-Bicknell C, et al (2003) GH/IGF-I independent genetic effects on BMD and skeletal morphology are both gender dependent and independent. Journal of Bone and Mineral Research 18: S123–S123.

[45] Gibson G (2009) Decanalization and the origin of complex disease. Nature Reviews Genetics 10: 134–140. 10.1038/nrg2502 19119265

[46] Gibson G, Dworkin I (2004) Uncovering cryptic genetic variation. Nature Reviews Genetics 5: 681–690. 10.1038/nrg1426 15372091

[47] Beamer W, Eicher EM (1976) Stimulation of growth in the little mouse. Journal of Endocrinology 71: 37–45. 10.1677/joe.0.0710037 978118

[48] Mathews LS, Norstedt G, Palmiter RD (1986) Regulation of insulin-like growth factor I gene expression by growth hormone. Proceedings of the National Academy of Sciences of the United States of America 83: 9343–9347. 10.1073/pnas.83.24.9343 3467309 PMC387134

[49] Blake JA, Bult CJ, Eppig JT, Kadin JA, Richardson JE, et al (2014) The Mouse Genome Database: integration of and access to knowledge about the laboratory mouse. Nucleic Acids Research 42: D810–D817. 10.1093/nar/gkt1225 24285300 PMC3964950

[50] Rosen CJ, Dimai HP, Vereault D, Donahue LR, Beamer WG, et al (1997) Circulating and skeletal insulin-like growth factor-I (IGF-I) concentrations in two inbred strains of mice with different bone mineral densities. Bone 21: 217–223. 10.1016/S8756-3282(97)00143-9 9276086

[51] Rosen CJ, Churchill GA, Donahue LR, Shultz KL, Burgess JK, et al (2000) Mapping quantitative trait loci for serum insulin-like growth factor-1 levels in mice. Bone 27: 521–528. 10.1016/S8756-3282(00)00354-9 11033447

[52] Schneider P, Stauber M, Voide R, Stampanoni M, Donahue LR, et al (2007) Ultrastructural properties in cortical bone vary greatly in two inbred strains of mice as assessed by synchrotron light based micro-and nano-CT. Journal of Bone and Mineral Research 22: 1557–1570. 10.1359/jbmr.070703 17605631

[53] MouseMine Mouse Genome Informatics Web Site. The Jackson Laboratory, Bar Harbor, Maine. World Wide Web (URL: http://www.mousemine.org/). Accessed: November, 2014.

[54] Keane TM, Goodstadt L, Danecek P, White MA, Wong K, et al (2011) Mouse genomic variation and its effect on phenotypes and gene regulation. Nature 477: 289–294. 10.1038/nature10413 21921910 PMC3276836

[55] Burgess-Herbert SL, Cox A, Tsaih SW, Paigen B (2008) Practical applications of the bioinformatics toolbox for narrowing quantitative trait loci. Genetics 180: 2227–2235. 10.1534/genetics.108.090175 18845850 PMC2600954

[56] Shockley KR, Witmer D, Burgess-Herbert SL, Paigen B, Churchill GA (2009) Effects of atherogenic diet on hepatic gene expression across mouse strains. Physiological Genomics 39: 172–182. 10.1152/physiolgenomics.90350.2008 19671657 PMC2789673

[57] Mesner LD, Ray B, Hsu YH, Manichaikul A, Lum E, et al (2014) *Bicc1* is a genetic determinant of osteoblastogenesis and bone mineral density. The Journal of Clinical Investigation 124: 2736–2749. 10.1172/JCI73072 24789909 PMC4038574

[58] Alon U (2007) Network motifs: theory and experimental approaches. Nature Reviews Genetics 8: 450–461. 10.1038/nrg2102 17510665

[59] Phillips PC (2008) Epistasis–the essential role of gene interactions in the structure and evolution of genetic systems. Nature Reviews Genetics 9: 855–867. 10.1038/nrg2452 18852697 PMC2689140

[60] Lehner B (2011) Molecular mechanisms of epistasis within and between genes. Trends in Genetics 27: 323–331. 10.1016/j.tig.2011.05.007 21684621

[61] Avery L, Wasserman S (1992) Ordering gene function: the interpretation of epistasis in regulatory hierarchies. Trends in Genetics 8: 312–316. 10.1016/0168-9525(92)90263-4 1365397 PMC3955268

[62] Segrè D, DeLuna A, Church GM, Kishony R (2005) Modular epistasis in yeast metabolism. Nature Genetics 37: 77–83. 15592468 10.1038/ng1489

[63] St Onge RP, Mani R, Oh J, Proctor M, Fung E, et al (2007) Systematic pathway analysis using high-resolution fitness profiling of combinatorial gene deletions. Nature Genetics 39: 199–206. 10.1038/ng1948 17206143 PMC2716756

[64] Cummings SR, Melton LJ (2002) Epidemiology and outcomes of osteoporotic fractures. The Lancet 359: 1761–1767. 10.1016/S0140-6736(02)08657-9 12049882

[65] Falahati-Nini A, Riggs BL, Atkinson EJ, O’Fallon WM, Eastell R, et al (2000) Relative contributions of testosterone and estrogen in regulating bone resorption and formation in normal elderly men. Journal of Clinical Investigation 106: 1553–1560. 10.1172/JCI10942 11120762 PMC381474

[66] Khosla S, Melton LJ III, Atkinson EJ, O’Fallon W (2001) Relationship of serum sex steroid levels to longitudinal changes in bone density in young versus elderly men. Journal of Clinical Endocrinology & Metabolism 86: 3555–3561. 10.1210/jcem.86.8.7736 11502778

[67] Alam I, Sun Q, Liu L, Koller DL, Carr LG, et al (2008) Sex-specific genetic loci for femoral neck bone mass and strength identified in inbred COP and DA rats. Journal of Bone and Mineral Research 23: 850–859. 10.1359/JBMR.080221 18282130 PMC2677085

[68] Turner CH, Sun Q, Schriefer J, Pitner N, Price R, et al (2003) Congenic mice reveal sex-specific genetic regulation of femoral structure and strength. Calcified Tissue International 73: 297–303. 10.1007/s00223-002-1062-1 14667144

[69] Gibson G, Wagner G (2000) Canalization in evolutionary genetics: a stabilizing theory? BioEssays 22: 372–380. 10.1002/(SICI)1521-1878(200004)22:4%3C372::AID-BIES7%3E3.3.CO;2-A 10723034

[70] Hartman JL, Garvik B, Hartwell L (2001) Principles for the buffering of genetic variation. Science 291: 1001–1004. 11232561 10.1126/science.1056072

[71] Mackay TFC (2014) Epistasis and quantitative traits: using model organisms to study gene-gene interactions. Nature Reviews Genetics 15: 22–33. 10.1038/nrg3627 24296533 PMC3918431

[72] Edwards AC, Mackay TFC (2009) Quantitative trait loci for aggressive behavior in Drosophila melanogaster. Genetics 182: 889–897. 10.1534/genetics.109.101691 19414563 PMC2710167

[73] Gale GD, Yazdi RD, Khan AH, Lusis AJ, Davis RC, et al (2009) A genome-wide panel of congenic mice reveals widespread epistasis of behavior quantitative trait loci. Molecular Psychiatry 14: 631–645. 10.1038/mp.2008.4 18379576 PMC3014058

[74] Shao H, Burrage LC, Sinasac DS, Hill AE, Ernest SR, et al (2008) Genetic architecture of complex traits: large phenotypic effects and pervasive epistasis. Proceedings of the National Academy of Sciences 105: 19910–19914. 10.1073/pnas.0810388105 PMC2604967 19066216

[75] Polderman T, Benyamin B, de Leeuw C, Sullivan P, van Bochoven A, et al (2015) Meta-analysis of the heritability of human traits based on fifty years of twin studies. Nature Genetics 47:702–709. 10.1038/ng.3285 25985137

[76] Ackert-Bicknell CL, Salisbury JL, Horowitz M, Demambro VE, Horton LG, et al (2007) A Chromosomal Inversion within a Quantitative Trait Locus Has a Major Effect on Adipogenesis and Osteoblastogenesis. Annals of the New York Academy of Sciences 1116: 291–305. 10.1196/annals.1402.010 17584978

[77] Beamer WG, Shultz KL, Churchill GA, Frankel WN, Baylink DJ, et al (1999) Quantitative trait loci for bone density in C57BL/6J and CAST/EiJ inbred mice. Mammalian Genome 10: 1043–1049. 10.1007/s003359901159 10556421

[78] Johnson KR, Marden CC, Ward-Bailey P, Gagnon LH, Bronson RT, et al (2007) Congenital Hypothyroidism, Dwarfism, and Hearing Impairment Caused by a Missense Mutation in the Mouse Dual Oxidase 2 Gene, Duox2. Molecular Endocrinology 21: 1593–1602. 10.1210/me.2007-0085 17440044

[79] Beamer WG, Donahue LR, Rosen CJ, Baylink DJ (1996) Genetic variability in adult bone density among inbred strains of mice. Bone 18:397–403. 10.1016/8756-3282(96)00047-6 8739896

[80] Adamo ML, Ma X, Ackert-Bicknell CL, Donahue LR, Beamer WG, et al (2006) Genetic increase in serum insulin-like growth factor-I (IGF-I) in C3H/HeJ compared with C57BL/6J mice is associated with increased transcription from the IGF-I exon 2 promoter. Endocrinology 147: 2944–2955. 10.1210/en.2005-0742 16527837

[81] Carter GW, Hays M, Sherman A, Galitski T (2012) Use of pleiotropy to model genetic interactions in a population. PLoS Genetics 8: e1003010 10.1371/journal.pgen.1003010 23071457 PMC3469415

[82] Broman KW, Wu H, Sen S, Churchill GA (2003) R/qtl: QTL mapping in experimental crosses. Bioinformatics 19: 889–890. 10.1093/bioinformatics/btg112 12724300

[83] Bevington P (1994) Data reduction and error analysis for the physical sciences. New York: McGraw-Hill.

[84] Tyler AL, McGarr TC, Beyer BJ, Frankel WN, Carter GW (2014) A genetic interaction network model of a complex neurological disease. Genes, Brains, Behavior 13:831–840. 10.1111/gbb.12178 PMC4241132 25251056

[85] Holm S (1979) A simple sequentially rejective multiple test procedure. Scandinavian Journal of Statistics 6: 65–70.

[86] Clauset A, Newman MEJ, Moore C (2004) Finding community structure in very large networks. Physical Review E 70: 066111 10.1103/PhysRevE.70.066111 15697438

[87] Csardi G, Nepusz T (2006) The igraph software package for complex network research. Complex Systems: 1695.

[88] Wong AK, Park CY, Greene CS, Bongo LA, Guan Y, et al (2012) Imp: a multi-species functional genomics portal for integration, visualization and prediction of protein functions and networks. Nucleic Acids Research 40: W484–W490. 10.1093/nar/gks458 22684505 PMC3394282

[89] R Core Team (2015) R: A Language and Environment for Statistical Computing. R Foundation for Statistical Computing, Vienna, Austria. URL http://www.R-project.org/

